# 小细胞肺癌的分子分型及其治疗策略的研究进展

**DOI:** 10.3779/j.issn.1009-3419.2023.106.08

**Published:** 2023-04-20

**Authors:** WU Yang, ZHAO Jing, WANG Mengzhao

**Affiliations:** ^1^100084 北京，清华大学医学院（吴杨）; ^1^School of Medicine, Tsinghua University, Beijing 100084, China; ^2^100730 北京，中国医学科学院，北京协和医学院，北京协和医院呼吸与危重症医学科（赵静，王孟昭）; ^2^Department of Pulmonary and Critical Care Medicine, Peking Union Medical College Hospital, Peking Union Medical College and Chinese Academy of Medical Sciences, Beijing 100730, China

**Keywords:** 肺肿瘤, 分子分型, 临床特征, 治疗, Lung neoplasms, Molecular subtype, Clinical characteristic, Treatment

## Abstract

小细胞肺癌（small cell lung cancer, SCLC）是一种具有极强增殖和侵袭能力的恶性肿瘤，由于缺乏有效治疗手段，临床预后差。近年来，对细胞、动物模型及肿瘤组织本身的研究推动了SCLC分子亚型的提出，并发现不同分子亚型的SCLC具有独特的生物学和临床特征及潜在的特异性治疗靶点，这将有助于为SCLC患者制定更精准的治疗策略，以期改善预后。本文拟对目前关于SCLC的分子分型进行综述，重点关注不同分子分型的SCLC的临床特征以及治疗策略，并提出未来SCLC治疗的合理建议。

## Abstract (en)

Small cell lung cancer (SCLC) is a malignant tumor with remarkable proliferative and invasive ability, which has very poor clinical prognosis due to lack of effective treatments. In recent years, researches on cells, animal models and tumor samples have promoted the identification of molecular subtypes of SCLC, discovered unique biological and clinical characteristics, and proposed potential specific therapeutic targets for different subtypes. This will encourage the development of more accurate therapeutic strategies towards SCLC, with a view to improving the prognosis of the patients. This article will review the current SCLC molecular subtypes, focus on the clinical characteristics and therapeutic strategies of different SCLC subtypes, and propose reasonable suggestions for the future treatment of SCLC.

## 1 背景

肺癌是世界范围内导致癌症相关死亡的首要原因，根据病理学特征被分为两类：小细胞肺癌（small cell lung cancer, SCLC）和非小细胞肺癌（non-small cell lung cancer, NSCLC）^[1⇓-3]^。其中SCLC约占肺癌总数的15%，以基因组不稳定、TP53和RB1基因失活、生长和侵袭能力强等为主要特征，整体预后极差^[4]^。SCLC曾被普遍认为是单一类型的同质化疾病，与可根据驱动基因突变选择靶向治疗的NSCLC不同，SCLC通常仅根据疾病的临床分期及复发情况来选择治疗方案，缺乏可靠的分子标志物指导分型和治疗^[5]^。但近来随着对SCLC细胞、动物模型及肿瘤组织研究的深入，不同亚型的SCLC被逐渐识别和鉴定出来。目前被广泛认可的分型模式是Rudin等^[6]^基于SCLC 81例肿瘤样本和54株细胞系的基因表达谱鉴定出的SCLC-A、SCLC-N、SCLC-P、SCLC-Y共4种分子亚型，分别以表达转录因子ASCL1、NEUROD1、POU2F3、YAP1为主要特征，其中SCLC-A和SCLC-N亚型为神经内分泌（neuroendocrine, NE）亚型，SCLC-P和SCLC-Y亚型为非神经内分泌（non-neuroendocrine, non-NE）亚型。SCLC分型的提出，对于推动SCLC的精准治疗具有重要意义，本文拟对不同SCLC分子亚型的生物学特征、临床特征、靶向治疗策略的研究进行综述，并对未来SCLC的治疗策略进行展望。

## 2 不同SCLC亚型的分子生物学特征

### 2.1 SCLC-A

ASCL1是建立肺神经内分泌细胞谱系所必需的转录因子^[7]^，在表达ASCL1的SCLC-A亚型中具有维持细胞存活及神经内分泌表型的作用。RNA干扰抑制ASCL1转录，可抑制细胞增殖能力并诱导细胞凋亡。基因表达分析显示ASCL1直接调控2个干细胞标记基因CD133和ALDH1A1的表达，赋予肿瘤生长能力^[8]^。基于转基因小鼠的研究^[9]^显示，ASCL1的缺失抑制了SCLC肿瘤的起始及发生，但促进了表达SOX9的神经嵴干细胞的增加以及骨肉瘤和软骨样肿瘤的出现，提示ASCL1对该分化途径具有抑制作用。

### 2.2 SCLC-N

转录因子NEUROD1对几种神经/神经内分泌组织的发育和功能、特定神经元的细胞分化方向以及成人胰腺β细胞中胰岛素基因转录起重要作用^[10,11]^。在SCLC-N亚型中，NEUROD1是维持肿瘤生存和迁移以及诱导相关信号通路的调节中枢，可上调原肌球蛋白相关激酶B（tropomyosin kinase B receptor, TrkB）和神经细胞黏附分子（neural cell adhesion molecule, NCAM）的表达，二者在生理状态下能调节神经元分化、细胞存活、轴突生长和迁移^[12,13]^，而在SCLC-N亚型中被NEUROD1激活后，可促进肿瘤的存活和转移^[14]^。此外，尽管ASCL1和NEUROD1都属于一类含螺旋-环-螺旋结构的转录因子，但由于二者在介导转录因子-DNA结合的识别序列方面的差异，它们实际结合的位点及调控的下游基因也大不相同^[15]^。

### 2.3 SCLC-P

POU2F3是一种在簇细胞中选择性表达的转录因子，簇细胞是呼吸道中一种罕见的化学感受细胞类型^[16]^。SCLC-P亚型肿瘤中缺乏经典NE标志物的表达，而高表达SOX9、ASCL2等一系列簇细胞标志物，且研究^[17]^证实其受POU2F3调控，意味着它可能和NE-SCLC不同起源，但SCLC-P亚型肿瘤仍具有与NE-SCLC相似的极高的增殖率。此外，在SCLC-P亚型中还发现另一个转录因子POU2AF2能改变染色体结构，并与POU2F3形成异二聚体，共同调控下游基因表达^[18]^。

### 2.4 SCLC-Y

YAP1所参与的Hippo信号通路被证明是许多肿瘤中发生恶性转化的主要调节因子，影响细胞增殖和干细胞的生长^[19]^。YAP1在其他实体瘤中广泛表达和激活，但在SCLC中只表达于少数non-NE表型的肿瘤^[20,21]^。在SCLC-Y亚型的肿瘤中，YAP1可通过Notch依赖性或非依赖性途径发出信号，通过诱导Rest表达促进SCLC从NE向non-NE肿瘤细胞的命运转换，YAP1的激活还可增强SCLC肿瘤细胞的化疗耐药性^[22]^。在SCLC-Y亚型中敲除YAP1降低了细胞增殖和侵袭能力，并恢复了药物敏感性和NE标志物的表达^[23]^。此外，SCLC-Y亚型细胞还表现出独特的形态学特征，即高表达层黏连蛋白和整合素的贴壁形态，敲除YAP1后改变为SCLC细胞常见的悬浮生长模式^[24]^。

## 3 不同SCLC亚型的临床特征

尽管在细胞和动物模型中已有大量关于SCLC亚型的研究^[6,15,17]^，但对于不同亚型的病理特点、免疫浸润、预后等临床特征的研究相对较少，且主要来自于小型队列的回顾性研究^[24⇓⇓-27]^，结论存在争议。早期对于SCLC细胞系的研究^[28]^发现，SCLC-A亚型的细胞系更常见于来自未治疗的患者，而SCLC-N亚型的细胞系则更可能来自于先前已治疗过的患者，推测这可能与SCLC-N亚型更适应化疗的选择压力有关。Qi等^[25]^发现与SCLC-Y亚型患者相比，SCLC-A亚型患者的脑转移或骨转移发生率更高，而SCLC-N亚型的患者肝转移发生率更高；免疫浸润方面，SCLC-Y亚型与更低的细胞毒性T淋巴细胞相关蛋白4（cytotoxic T-lymphocyte-associated protein 4, CTLA-4）表达相关，而SCLC-A亚型与更高的叉头盒蛋白P3（forkhead box protein P3, FOXP3）、程序性死亡受体1（programmed cell death 1, PD-1）和CTLA-4表达相关。

此外，SCLC亚型分类被确定为预测SCLC肿瘤生存结局的独立指标。SCLC-Y亚型患者表现出最长的总生存期（overall survival, OS）和无进展生存期（progression-free survival, PFS）^[25]^。相似的是，Owonikoko等^[24]^发现YAP1蛋白更显著地在局限期SCLC中富集，且SCLC-Y亚型与干扰素-γ应答基因的高表达、T细胞激活基因高表达相关，均表明SCLC-Y亚型预后良好。Furuta等^[27]^发现在接受手术治疗的早期SCLC患者中，ASCL1高表达与纯SCLC（pure SCLC, P-SCLC）的组织学特征显著相关，也显示更低的OS。但与前面研究相矛盾的是，McColl等^[26]^利用INSM1/YAP1的相对表达比对SCLC患者进行分层，该比值高的SCLC-A和SCLC-N亚型与比值低的SCLC-Y亚型相比，展示出更长的OS和PFS。Wang等^[29]^发现YAP1在混合型SCLC（combined SCLC, C-SCLC）中阳性率显著高于P-SCLC，且YAP1的表达与较差的OS相关，在C-SCLC中是OS的独立危险因素。Song等^[30]^报道YAP1能在体内及体外介导SCLC对多种化疗药物的耐药性，YAP1高表达提示更短的OS和更晚期的疾病阶段。综上，目前关于SCLC亚型与临床预后的关系尚不明确，需要在更大队列的前瞻性研究中进一步探索。

## 4 基于不同SCLC亚型的靶向治疗策略

### 4.1 SCLC-A

针对SCLC-A亚型的潜在靶点主要包括受转录因子ASCL1调控的下游蛋白DLL3和BCL2以及与组蛋白修饰相关的赖氨酸特异性组蛋白去甲基化酶（lysine specific demethylase 1, LSD1）和CREB结合蛋白（CREB binding protein, CREBBP）。DLL3是一种非典型Notch受体家族配体，在部分NE-SCLC细胞的表面特异性表达，由靶向DLL3抗体耦合细胞毒性药物的Rova-T（Rovalpituzumab tesirine）在SCLC的二线治疗中展现出一定的临床获益^[31]^，其余靶向DLL3的治疗还包括双特异性T细胞接合蛋白和嵌合抗原受体T细胞治疗^[32]^。而研究^[33]^发现DLL3在SCLC中的表达与ASCL1呈正相关，意味着SCLC-A亚型可能因为高表达DLL3，而对基于DLL3的治疗有更高的敏感性。与DLL3类似，BCL2也在SCLC-A亚型中高表达并发挥抗细胞凋亡的作用^[34]^，多种BCL2抑制剂在体内和体外实验中都表现出对SCLC-A肿瘤生长有很好的抑制效果^[35]^。LSD1抑制剂T-3775440被报道可通过破坏LSD1和SNAG域转录因子INSM1之间的相互作用，从而抑制SCLC的神经内分泌相关转录和细胞增殖活性，这一效应与NE亚型的SCLC-A和SCLC-N相关^[36]^。此外，另一个LSD1抑制剂ORY-1001可以激活Notch通路，进而抑制转录因子ASCL1的活性及肿瘤生长^[37]^。基于SCLC转基因小鼠模型的研究^[38]^发现，组蛋白乙酰转移酶相关CREBBP的失活可能驱动SCLC-A亚型肿瘤的发展，而组蛋白脱乙酰酶抑制剂Pracinostat可以抑制这一过程，体现出潜在的疗效。

### 4.2 SCLC-N

SCLC-N亚型通常表现出MYC扩增的特征，靶向MYC及其相关通路是许多治疗策略研发核心，除直接特异性抑制MYC外^[39]^，由MYC驱动的肿瘤所展现出的其他独特性质，比如对Aurora激酶抑制剂的敏感性、对精氨酸的依赖性、高糖酵解活性、高嘌呤途径代谢物活性等^[40⇓-42]^，也可作为有价值的治疗靶点。靶向TrkB及其他酪氨酸激酶抑制剂来他替尼可显著抑制由NEUROD1下游TrkB和NCAM介导的肿瘤增殖和迁移^[14]^。此外，NEUROD1和ASCL1的应答基因的启动子周围显示出开放的染色质结构，DNA结合剂Lurbinectedin能结合位于激活基因转录起始点下游的富含CpG的区域，抑制下游基因表达并诱导细胞凋亡^[43]^。值得一提的是，SCLC-N亚型表现出对塞内卡山谷病毒（Seneca Valley virus, SVV）独特的易感性，该溶瘤病毒能选择性识别并感染SCLC-N亚型的肿瘤细胞，并通过裂解清除，NEUROD1与ASCL1的表达比值还可作为生物标志物预测SVV的疗效^[44]^。通过适当的生物标志物指导患者选择，SVV溶瘤病毒可能作为单剂治疗或与免疫疗法联合使用展现选择性疗效^[45]^。

### 4.3 SCLC-P

一项基于激酶结构域的CRISPR筛选发现SCLC-P亚型对胰岛素样生长因子1受体（insulin-like growth factor 1 receptor, IGF-1R）具有独特的依赖性，这提示以达罗托组单抗为代表的IGF-1R抑制剂可能作为这些患者的潜在特异性治疗手段^[17]^。Gay等^[46]^对SCLC细胞系进行了广泛的药物筛选试验，其中SCLC-P亚型对聚腺苷酸二磷酸核糖聚合酶（poly ADP-ribose polymerase, PARP）抑制剂展现出最强的敏感性，这种敏感性也体现在核苷类似物和抗叶酸药物中。通常对PARP抑制剂的敏感性与SLFN11高水平表达相关^[47,48]^，但也有研究^[46]^显示，SCLC-P亚型对其的敏感性似乎并不依赖于SLFN11。

### 4.4 SCLC-Y

免疫检查点抑制剂（immune checkpoint inhibitor, ICI）已被指南推荐联合依托泊苷作为广泛期SCLC的一线治疗方案，但PD-1/程序性死亡配体1（programmed cell death ligand 1, PD-L1）的表达在SCLC中有很大的异质性^[49]^，不同SCLC亚型对ICI反应也可能不同。就SCLC-Y亚型而言，YAP1可能通过上调PD-L1表达，募集M2巨噬细胞、髓源性抑制细胞、调节性T细胞，抑制自然杀伤（natural killer, NK）细胞等途径营造免疫抑制环境^[50]^，且SCLC-Y肿瘤细胞具有更高的CD38和LAG-3表达水平，因此该亚型可能对免疫检查点抑制剂具有更好的治疗反应^[45]^。此外基于转录组水平的分析结果显示，SCLC-Y亚型对哺乳动物雷帕霉素靶蛋白（mammalian target of rapamycin, mTOR）、丝氨酸/苏氨酸蛋白激酶（polo-like kinase, PLK）和细胞周期蛋白依赖性激酶4/6（cyclin-dependent kinase 4/6, CDK4/6）抑制剂也展示出更高的敏感性^[51]^。

### 4.5 SCLC靶向治疗策略的临床试验结果

尽管上述一些针对具体SCLC亚型的靶向治疗策略展现出很好的临床应用前景，但它们主要是基于细胞系和动物模型的实验数据，尚缺乏相应的临床试验结果的报道。目前已有靶向治疗的试验结果的报道主要是针对未分型的整体SCLC患者。一项II期临床研究^[52]^发现替莫唑胺加PARP抑制剂维利帕尼相比替莫唑胺加安慰剂显著提高复发性SCLC患者的客观缓解率（objective response rate, ORR），ORR为39% vs 14%（P=0.016），但两组的中位OS（8.2个月 vs 7.0个月，P=0.50）和PFS（3.9个月 vs 2.0个月，P=0.39）没有显著差异。ALTER 1202研究^[53]^发现三线治疗应用多靶点酪氨酸激酶抑制剂安罗替尼相比对照组能显著改善二线化疗失败SCLC患者的中位OS（7.3个月 vs 4.9个月，P=0.0029）和PFS（4.1个月 vs 0.7个月，P<0.0001）。AURKA抑制剂Alisertib作为二线治疗相比对照组尽管不能显著改善整体患者的中位PFS（3.3个月 vs 2.2个月，P=0.113），但在携带CDK6/RB1/RBL1/RBL2基因突变的亚组患者中效果显著（3.7个月 vs 1.8个月，P=0.0003）^[54]^。TRINITY研究^[55]^发现在三线及以上治疗中应用靶向DLL3抗体耦联药物Rova-T的ORR为12.4%，其中ORR在DLL3表达阳性患者中也仅为13.2%，但3级以上的副作用却出现在63%的患者中。IGF-1R抑制剂Linsitinib相比托泊替康并未展现出中位OS的提升（3.4个月 vs 5.3个月，P=0.71），中位PFS甚至劣于后者（1.2个月 vs 3.0个月，P=0.0001）^[56]^。上述研究结果有一定参考意义，但仍需要针对特定亚型设计临床试验，以获得高质量的临床证据来支持基于亚型的靶向治疗策略。

## 5 SCLC分型的争议与挑战

尽管上述基于4种转录因子ASCL1、NEUROD1、POU2F3、YAP1表达水平定义的SCLC分型模式已被广泛报道和使用，但该模式能否全面准确地反映SCLC分型的实际情况仍存在争议，如有两项研究^[57,58]^在分别对174例原位肿瘤和39例循环肿瘤细胞移植物样本做免疫组织化学染色后，均未检测到以独特表达YAP1为特征的SCLC亚型。Gay等^[46]^进一步提出，与SCLC-A、SCLC-N、SCLC-P这3种以受特定转录因子调控为主要特征的亚型不同的是，第4种亚型并非受某特定转录因子调控，而是表达许多免疫检查点和人类白细胞抗原相关基因，被定义为一类炎症亚型SCLC（SCLC-inflamed, SCLC-I），也推测SCLC-I亚型的患者在ICI治疗下有更好的预后。有趣的是，Qu等^[59]^提出了5种亚型的分型模式，他们在146例SCLC原位肿瘤中再次证实了SCLC-A、SCLC-N、SCLC-P、SCLC-Y这4种亚型的存在，但也观察到第5种亚型不表达前4种转录因子，但展现出与SCLC-I亚型相似的炎症激活背景。关于SCLC-Y亚型存在与否的矛盾可能是出于以下2个原因：（1）SCLC-Y亚型是4种经典SCLC亚型中所占比例最少的一种，本身相对少见，在样本量少的情况下较难被捕捉到^[59]^；（2）YAP1的表达还与肿瘤阶段相关，如Owonikoko等^[24]^提出YAP1更加在局限期SCLC中富集，而此前未发现SCLC-Y亚型的研究所选样本均来源于进展期SCLC。但共同之处在于，SCLC-Y与SCLC-I亚型都显示出独特的免疫浸润环境，并可能提示更好的免疫治疗反应。

除上述关于SCLC-Y、SCLC-I亚型的争议外，SCLC肿瘤的空间和时间异质性也给SCLC分型模式进一步带来挑战，即同一SCLC肿瘤内可能同时存在多种亚型，且亚型之间会发生相互转化。比如Borromeo等^[15]^和Mollaoglu等^[40]^都发现SCLC-A亚型是SCLC-N亚型的必要前体，各SCLC亚型之间存在层级关系。Stewart等^[60]^将复发SCLC来源的循环肿瘤细胞移植物与对应的初治SCLC肿瘤样本相比较，发现了肿瘤异质性在经过治疗后进一步增加，并可能为介导化疗耐药性的机制之一。单细胞水平的测序手段的应用，能实现对复杂的肿瘤微环境中包括肿瘤细胞、免疫细胞、基质细胞等所有细胞类型的区分，并鉴定这些多细胞成分之间的相互作用，有助于更加全面地解析SCLC肿瘤内异质性和生物可塑性^[61,62]^。最近对SCLC肿瘤进行单细胞测序的研究^[63,64]^更加精准地显示了同一肿瘤中不同亚型的细胞所占的具体比例，甚至捕捉到了一小部分介于SCLC-A和SCLC-N亚型之间的中间态细胞，明确提供了亚型动态转化的证据。

## 6 总结

准确定义SCLC亚型并了解不同亚型的生物学和临床特征，找到亚型特有的治疗靶点，可以为SCLC的精准治疗提供基础。未来目前分型模式的准确性有待进一步校正和完善，同时肿瘤异质性也不容忽视，需要用单细胞测序等手段来实现更加精准动态的监测，以期为SCLC患者制定出更加有针对性的个体化治疗方案，提高疗效，改善预后。
